# Vasoactive-ventilation-renal score and outcomes in infants and children after cardiac surgery

**DOI:** 10.3389/fped.2023.1086626

**Published:** 2023-02-20

**Authors:** Pota Abhay, Rajesh Sharma, Anil Bhan, Manan Raina, Ananya Vadhera, Romel Akole, Firdoos Ahmad Mir, Pankaj Bajpai, Amit Misri, Swarnika Srivastava, Ved Prakash, Tanmoy Mondal, Anvitha Soundararajan, Abhishek Tibrewal, Shyam Bihari Bansal, Sidharth Kumar Sethi

**Affiliations:** ^1^ Pediatric Cardiology, Medanta, The Medicity Hospital, Gurgaon, India; ^2^ Pediatric Cardiac Intensive Care, Medanta—The Medicity, Gurgaon, India; ^3^ CTVS, Medanta—The Medicity, Gurgaon, India; ^4^ Hawken High School, Cleveland, OH, United States; ^5^ Maulana Azad Medical College, New Delhi, India; ^6^ Cardiology, Medanta—The Medicity, Gurgaon, India; ^7^ Akron Nephrology Associates, Akron General Cleveland Clinic, Akron, OH, United States; ^8^ Pediatric Nephrology, Akron's Children Hospital, Akron, OH, United States; ^9^ Kidney Institute, Medanta, The Medicity, Gurgaon, India

**Keywords:** pediatrics, cardiac surgery, vasoactive-ventilation-renal score, PICU, infants

## Abstract

**Introduction:**

There is a need to index important clinical characteristics in pediatric cardiac surgery that can be obtained early in the postoperative period and accurately predict postoperative outcomes.

**Methodology:**

A prospective cohort study was conducted in the pediatric cardiac ICU and ward on all children aged <18 years undergoing cardiac surgery for congenital heart disease from September 2018 to October 2020. The vasoactive-ventilation-renal (VVR) score was analyzed to predict outcomes of cardiac surgeries with a comparison of postoperative variables.

**Results:**

A total of 199 children underwent cardiac surgery during the study period. The median (interquartile range) age was 2 (0.8–5) years, and the median weight was 9.3 (6–16) kg. The most common diagnoses were ventricular septal defect (46.2%) and tetralogy of Fallot (37.2%). At the 48th h, area under the curve (AUC) (95% CI) values were higher for the VVR score than those for other clinical scores measured. Similarly, at the 48th h, AUC (95% CI) values were higher for the VVR score than those for the other clinical scores measured for the length of stay and mechanical ventilation.

**Discussion:**

The VVR score at 48 h postoperation was found to best correlate with prolonged pediatric intensive care unit (PICU) stay, length of hospitalization, and ventilation duration, with the greatest AUC-receiver operating characteristic (0.715, 0.723, and 0.843, respectively). The 48-h VVR score correlates well with prolonged ICU, hospital stay, and ventilation.

## Introduction

Cardiac surgery in children is associated with longer recovery periods, increased need for hemodynamic support, longer duration of mechanical ventilation, and higher postoperative mortality and morbidity (1–8).

The potential adverse outcomes have been well defined; however, identifying high-risk patients with a poor prognosis is difficult due to the difference in anatomy and pathophysiology. This necessitates the development of indices that can be easily obtained early in the postoperative period and accurately predict postoperative outcomes.

The Wernovsky score was used to measure illness severity even though it was not designed for that purpose (9). However, further studies were unable to prove a correlation. Gaies et al. further developed this score by incorporating some additional medications to give the vasoactive-inotropic score (VIS) (10). Miletic et al. recently developed a new score, the vasoactive-ventilation-renal (VVR) score, to address the inability of previous scores to reflect multiorgan failure, especially concerning pulmonary and renal systems (11). The VVR score has been found to be easy to use, straightforward to calculate, and a strong predictor of postcardiac surgery outcomes (12). The VVR score was subsequently validated to predict postoperative outcomes and mortality in the pediatric postoperative cardiac surgical population (11, 13–15). Miletic et al. also found that the 48-h VVR score outperformed VIS and peak postoperative lactate in predicting postcardiac surgery outcomes. Similar findings validating the 48-h VVR score were seen in other studies (13, 16–18). Postcardiac surgery, these indices are useful in determining the severity of the patient's condition and the degree of support they require (11).

Other simpler postoperative monitoring parameters that may show postoperative outcomes include serum lactate (19, 20), risk adjustment for congenital heart surgery (RACHS)-1 (21), Society of Thoracic Surgeons—European Association for Cardio-thoracic Surgery (STAT) score (22), cross-clamp and bypass time (23), and high peak inspiratory pressure (PIP) and/or positive end-expiratory pressure (PEEP) requirement. Although several studies have demonstrated that lactate elevation is a predictor of postcardiac surgery outcomes, these studies differ in lactate cutoff values and types of surgical procedures. Thus, further studies are required to clarify the correlation between lactate levels and the corresponding outcomes (24, 25). While RACHS is a commonly used scoring system, it does not take into account details regarding the clinical status of the patient in the ICU. Similarly, STAT does not consider the clinical status and is focused on the risk of mortality rather than end-organ failure (26). Although VIS takes into account the clinical status, studies have demonstrated that its prediction is modest (10, 11). As per a study by Mehmood et al., cross-clamp and bypass time are not associated with prolonged mechanical ventilation and length of hospital stay (LOS). They demonstrated that various confounding factors play a bigger role in determining outcomes. Thus, cross-clamp and bypass time may not be as reliable a predictor of pediatric cardiac surgery outcomes as previously thought, and larger studies on the same are required (27). While these indices are valuable, the VVR score is a more comprehensive measure of postcardiac surgery outcomes.

We aimed to assess whether the VVR score can predict outcomes of cardiac surgeries in pediatric patients by focusing on the full repair of three of the most common congenital heart diseases, namely, atrial septal defect, ventricular septal defect, and tetralogy of Fallot. We further sought to compare the relatively complex VVR score with other simpler postoperative monitoring parameters such as RACHS-1 and STAT scores, cross-clamp time, bypass time, C-reactive protein (CRP), lactate, and high PIP and/or PEEP requirement and observe whether they fare reasonably well in their predictive ability.

## Materials and Methods

### Study type

This was a prospective cohort study.

### Study site

The study was conducted in the pediatric cardiac ICU and ward at a tertiary hospital.

### Study population

The study population included all patients below 18 years of age with congenital heart disease (atrial septal defect, ventricular septal defect, and tetralogy of Fallot) undergoing cardiac surgery.

### Inclusion criteria

The inclusion criteria were all patients below 18 years of age with congenital heart disease (atrial septal defect, ventricular septal defect, and tetralogy of Fallot) undergoing elective cardiac repair surgery.

### Exclusion criteria

The exclusion criteria were patients undergoing off-pump surgeries. Patients with long-standing underlying comorbidities were also excluded.

### Study duration

The duration of the study was from September 2018 to December 2020.

### Consent

All included patients were inpatients and were enrolled in the study after obtaining informed written consent.

### Outcomes

– Primary outcomes: The primary outcome measure was the total PICU stay in hours (for those who were admitted to the ICU more than once, the total duration of ICU stay was considered).
– Secondary outcomes: The secondary outcome measures were the length of stay in hours, total duration of inotropes in hours, and mechanical ventilation duration in hours (for those who were ventilated more than once, the total duration of mechanical ventilation was considered).

### Clinical data collection

After taking the basic patient profile, short clinical history, anthropometry, vitals, and systemic examination, preoperative investigations were performed according to the protocol of our institution. Data were accessed from patient files and PICU monitoring charts. All surgeries were performed under standard cardiopulmonary bypass through a median sternotomy by one primary cardiac surgeon and his surgical team. Perioperative data included age, weight, sex, cardiac diagnosis, presence of sepsis, cardiopulmonary bypass (CPB) and cross-clamp duration, pre-op creatinine value, RACHS-1 score, and STAT score. Postoperatively, ventilator settings, arterial blood gas (ABG), complete blood count (CBC), and renal function test (RFT) values, and inotrope doses were recorded at 1 h, 24 h, and 48 h after surgery. All patients in the postoperative period were assessed through recovery parameters (total ventilation duration, total length of ICU stay, inotrope requirement as calculated by vasoactive-ventilation-renal score at 24 and 48 h, total length of hospital stay).

### Score calculations

The VVR score was calculated at 1 h, 24 h, and 48 h after surgery, as follows: VIS + ventilation index (VI) + renal score (change in serum creatinine from baseline × 10).

VIS was calculated using the following equation:

Dopamine dose (μg/kg/min) + Dobutamine dose (μg/kg/min) + 100 × Epinephrine dose (μg/kg/min) + 10 × Milrinone dose (μg/kg/min) + 10,000 × Vasopressin dose (μg/kg/min) + 100 × Norepinephrine dose (μg/kg/min) (7).

VI was calculated using the following formula:

VI = respiratory rate (RR) × (PIP − PEEP) × PaCO_2_/1,000; ΔCr was calculated by subtracting serum creatinine (in mg/dL) at the time of each measurement from the preoperative serum creatinine and VVR using the following formula:

VVR = VIS + VI + (ΔCr × 10) (8).

For patients whose postoperative serum creatinine values were less than preoperative values, ΔCr was taken as 0. For patients not requiring ventilator support at the time of measurement, VI was taken as 0.

### Statistical analysis

All statistical analyses were performed using SPSS 21 software (IBM Corp). Median with interquartile range (IQR) was used to describe continuous data, whereas absolute count with percentage was used for categorical data. The outcomes of interest were PICU stay, length of hospital stay, and ventilation duration. These outcomes were dichotomized as the upper (worst) 25th percentile vs. lower (best) 75th percentile. The subjects in the upper 25th percentile were considered as having prolonged outcome. Univariate analysis was performed for demographic and clinical characteristics of patients to predict the outcomes using the Mann–Whitney *U* test, chi-square test, or Fisher exact test as appropriate for individual variables. Significance variables were included in the multivariate logistic regression model, and the odds ratio (OR) was calculated. *p* < 0.05 was considered significant.

The comparison across the different indexes was based on three main analyses. Data were analyzed for correlation between the scores and outcomes using Spearman's rho. Area under the curve (AUC) values for the outcomes were generated for different demographic and clinical variables of patients. Analysis of the discriminatory ability of VIS and VVR (at different time points) methods was performed using the C statistic comparison with receiver operating characteristic (ROC) curves of the two methods. The best cutoff value for the VVR score was derived having maximum accuracy and minimal weighted error.

## Results

This study included 199 children undergoing full repair of congenital heart defects that met the inclusion criteria. The median (IQR) age of the children was 2 (0.8–5) years, and the median weight was 9.3 (6–16) kg. Among the conditions evaluated, the most common diagnoses were ventricular septal defect (46.2%) and tetralogy of Fallot (37.2%). A RACHS-1 score of ≥3 and a STAT score of ≥2 were observed in 15.1% and 33.7%, respectively. At the 24th h, the median (IQR) VIS and VVR score were 7 (4–11) and 14.7 (7.2–23.4), respectively. At the 48th h, the median (IQR) VIS and VVR score were 4 (1–6) and 4 (1–7), respectively. The median (IQR) PICU stay was 80 (69.5–96.5) h, the length of hospital stay was 8 (7–9) days, and the ventilation duration was 16 (14–20) h. There were no cases of infection or death in the current study. The demographic and clinical profile of the subjects is given in Supplementary Table S1.

### PICU stay

**Figure 1:**
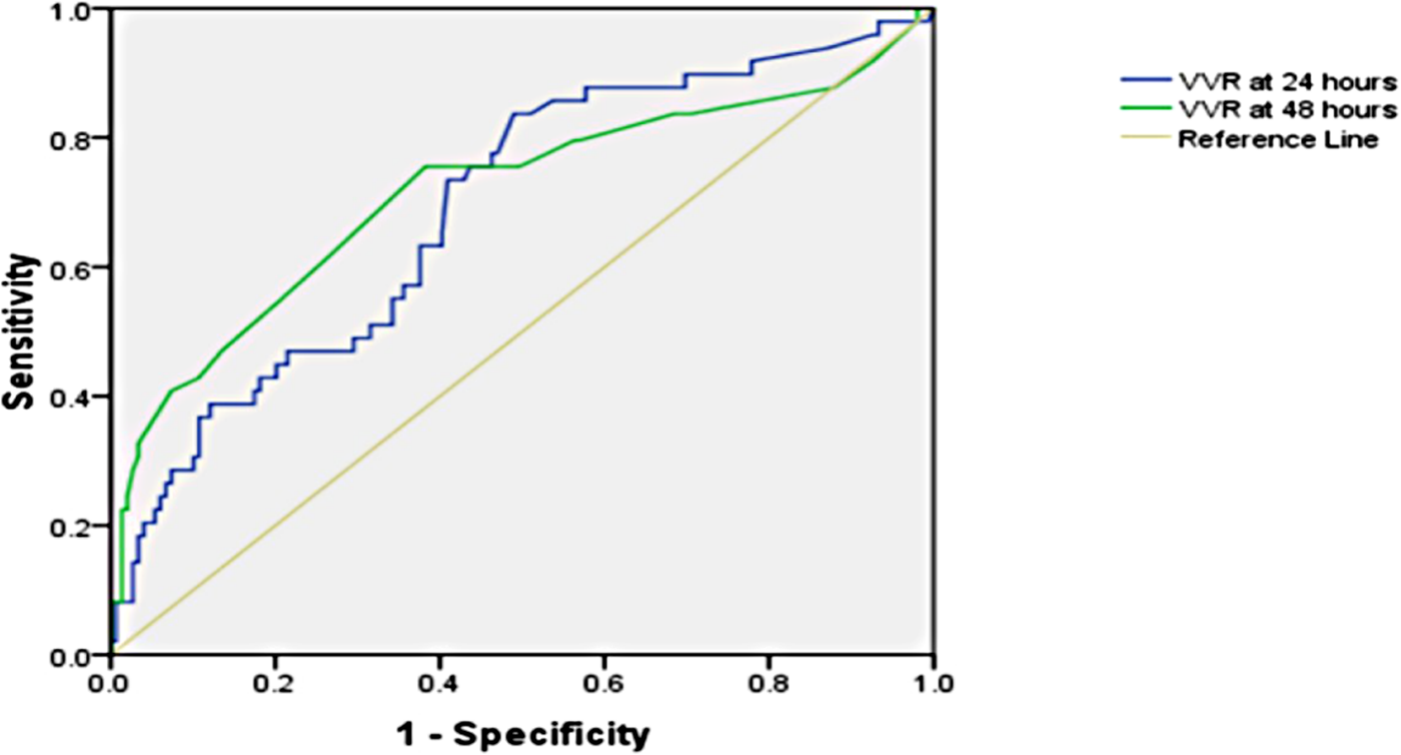
Receiver operating characteristic curve for the prolonged PICU stay and vasoactive-ventilation-renal score at 24 h and 48 h postoperatively.

The significant factors associated with prolonged PICU length of stay (>96.5 h) are age ≤1 year, bypass time, inotrope need at 48 h, preoperative oxygen saturation (SpO_2_), lactate at 48 h, respiratory rate at 24 h, peak inspiratory pressure at 24 h, peak inspiratory pressure at 48 h, positive end-expiratory pressure at 48 h, renal score at 48 h, VIS at 24 h, VIS at 48 h, VVR score at 24 h, and VVR score at 48 h. The results of the factors associated with the prolonged PICU stay [>75th percentile, i.e., 96.5 h], length of hospital stay, and ventilation duration are presented in Tables 1–3. When the multivariate regression analysis of the factors with *p* < 0.05 was done, age ≤1 year, preoperative SpO_2_, and the 48th-h VVR score were found to be independent risk factors. For prolonged PICU stay, at the 24th hour, AUC (95% CI) values were higher for the VVR score (*p* < 0.001) (Figure 1) than those for VI, renal score, and VIS. Similarly, at the 48th h, AUC (95% CI) values were higher for the VVR score (*p* < 0.001) (Figure 1) than those for VI, renal score, and VIS. The AUC values for none of the variables were superior to those for the 24th- or 48th-h VVR score in predicting the outcome (Table 4). No significant difference was found in the AUC values for 24th- and 48th-h VVR scores (z = 0.76; *p* = 0.446); however, at the 48th h, the AUC value for the VVR score was significantly better than that for the VIS (z = 2.49; *p* = 0.013) in predicting prolonged PICU stay. Also, in the correlation analysis, the VVR score predicted the outcome better than the VIS at each measurement point (Table 5). The best cutoff value of 4.6 for the VVR score at 48 h had a sensitivity of 76% and a specificity of 62% for prolonged PICU stay.

**Table 1:** Univariate analysis showing factors associated with prolonged PICU stay (>96.5 h).

Variables	>75th percentile (*n* = 49)	≤75th percentile (*n* = 149)	*p* value	OR (95% CI)
Age (≤1 year)	28 (57.1%)	43 (28.9%)	<0.001	3.29 (1.69–6.41)
Gender (male)	35 (71.4%)	99 (66.4%)	0.517	1.26 (0.62–2.56)
Diagnosis (TOF)	22 (44.9%)	51 (34.2%)	0.179	1.57 (0.81–3.02)
RACHS-1 (score ≥3)	10 (20.4%)	20 (13.4%)	0.237	1.65 (0.71–3.83)
STAT (score ≥2)	21 (42.9%)	45 (30.2%)	0.103	1.73 (0.89–3.37)
Cross-clamp time (min)^a^	47 (36.5–55.5)	43 (31.5–53.5)	0.141	NA
Bypass time (min)^a^	66 (56.5–77.5)	62 (50–71.5)	0.028	NA
Inotrope at 24 h (yes)	48 (98%)	134 (89.9%)	0.126	5.37 (0.69–41.78)
Inotrope at 48 h (yes)	48 (98%)	129 (86.6%)	0.025	7.44 (1.07–56.98)
Pre-op SpO_2_ (%)^a^	98 (92.5–100)	99 (98–100)	0.014	NA
Pre-op creatinine (mg/dL)^a^	0.3 (0.2–0.3)	0.3 (0.2–0.4)	0.264	NA
Creatinine at 24 h (mg/dL)^a^	0.4 (0.3–0.5)	0.4 (0.3–0.5)	0.411	NA
Creatinine at 48 h (mg/dL)^a^	0.4 (0.3–0.5)	0.4 (0.3–0.5)	0.173	NA
Pre-op CRP (mg/l)^a^	5 (5–5)	5 (5–5)	0.246	NA
Lactate at 1 h (mmol/L)^a^	2.64 (1.88–3.6)	2.3 (1.79–3.26)	0.122	NA
Lactate at 24 h (mmol/L)^a^	1.52 (1.12–2.2)	1.3 (1.03–1.8)	0.049	NA
Lactate at 48 h (mmol/L)^a^	1.23 (0.93–1.63)	1.08 (0.87–1.3)	0.023	NA
PCO_2_ at 24 h (mmHg)^a^	36.7 (32.1–40.8)	37.3 (34.3–40.1)	0.728	NA
PCO_2_ at 48 h (mmHg)^a^	38.7 (34.6–41.8)	37.8 (35.3–40.4)	0.235	NA
RR at 24 h^a^	30 (0–35)	16 (0–25)	<0.001	NA
RR at 48 h^a^	16 (0–25)	14 (0–22)	0.087	NA
PIP at 24 h^a^	17 (0–20)	14 (0–18)	0.007	NA
PIP at 48 h^a^	0 (0–0)	0 (0–0)	<0.001	NA
PEEP at 48 h^a^	0 (0–0)	0 (0–0)	<0.001	NA
PEEP at 24 h^a^	5 (0–5)	5 (0–5)	0.056	NA
Ventilation index at 24 h^a^	22.7 (0–154.51)	22.3 (0–111.25)	0.201	NA
Ventilation index at 48 h^a^	0 (0–0)	0 (0–0)	0.10	NA
Renal score at 24 h^a^	0.1 (0–0.2)	0.1 (0–0.1)	0.858	NA
Renal score at 48 h^a^	0.1 (0–0.2)	0.1 (0–0.1)	0.003	NA
VIS at 24 h^a^	12 (4–14)	7 (4–10)	0.001	NA
VIS at 48 h^a^	6 (1–10.5)	3 (1–5)	0.002	NA
VVR at 24 h^a^	19 (13.66–31.29)	12.79 (6–20.8)	<0.001	NA
VVR at 48 h^a^	7 (4–15)	3 (1–6)	<0.001	NA

TOF, tetralogy of Fallot; RACHS-1, risk adjustment for congenital heart surgery; STAT, Society of Thoracic Surgeons—European Association for Cardio-thoracic Surgery; SpO_2_, oxygen saturation; Pre-op, preoperative; CRP, C-reactive protein; PCO_2_, partial pressure of carbon dioxide; RR, respiratory rate; PIP, peak inspiratory pressure; PEEP, positive end-expiratory pressure; VIS, vasoactive inotrope score; VVR, vasoactive-ventilation-renal; OR, odds ratio; NA, not applicable.

^a^ Reported as median (IQR); others are reported as n (%).

NA for continuous variables as OR values are applicable for categorical variables only.

**Table 3:** Univariate analysis showing factors associated with prolonged ventilation duration (>20 h).

Variables	>75th percentile (*n* = 33)	≤75th percentile (*n* = 166)	*p* value	OR (95% CI)
Age (≤1 year)	22 (66.7%)	49 (29.5%)	<0.001	4.78 (2.15–10.6)
Gender (male)	22 (66.7%)	112 (67.5%)	0.928	0.96 (0.44–2.13)
Diagnosis (TOF)	16 (48.5%)	58 (34.9%)	0.141	1.75 (0.83–3.72)
RACHS-1 (score ≥3)	10 (30.3%)	20 (12%)	0.014	3.17 (1.32–7.63)
STAT (score ≥2)	15 (45.5%)	52 (31.3%)	0.117	1.83 (0.86–3.91)
Cross-clamp time (min)^a^	48 (39–58.5)	43.5 (32.75–53)	0.061	NA
Bypass time (min)^a^	65 (57.5–80)	62 (50–72)	0.073	NA
Inotrope at 24 h (yes)	33 (100%)	150 (90.4%)	0.079	Can't calculate
Inotrope at 48 h (yes)	33 (100%)	145 (87.3%)	0.028	Can't calculate
Pre-op SpO_2_ (%)^a^	98 (92–100)	99 (98–100)	0.004	NA
Pre-op creatinine (mg/dL)^a^	0.3 (0.2–0.35)	0.3 (0.2–0.4)	0.579	NA
Creatinine at 24 h (mg/dL)^a^	0.4 (0.3–0.5)	0.4 (0.3–0.5)	0.247	NA
Creatinine at 48 h (mg/dL)^a^	0.4 (0.3–0.5)	0.4 (0.3–0.5)	0.749	NA
Pre-op CRP (mg/L)^a^	5 (5–5)	5 (5–5)	0.069	NA
Lactate at 1 h (mmol/L)^a^	2.25 (1.72–3.26)	2.39 (1.8–3.39)	0.526	NA
Lactate at 24 h (mmol/L)^a^	1.5 (1.18–2.27)	1.35 (1.03–1.8)	0.067	NA
Lactate at 48 h (mmol/L)^a^	1.24 (0.93–1.46)	1.07 (0.89–1.32)	0.079	NA
PCO_2_ at 24 h (mmHg)^a^	37.7 (31.4–40.9)	37.1 (34.5–40.0)	0.631	NA
PCO_2_ at 48 h (mmHg)^a^	38 (34.5–41.1)	38 (35.8–40.8)	0.826	NA
RR at 24 h^a^	30 (9–40)	15 (0–25)	<0.001	NA
RR at 48 h^a^	20 (0–28)	14.5 (0–22)	0.036	NA
PIP at 24 h^a^	18 (8–20.5)	14 (0–18)	0.002	NA
PIP at 48 h^a^	0 (0–17)	0 (0–0)	<0.001	NA
PEEP at 48 h^a^	0 (0–5)	0 (0–0)	<0.001	NA
PEEP at 24 h^a^	5 (2.5–5)	5 (0–5)	0.012	NA
Ventilation index at 24 h^a^	19.1 (4.4–153.5)	54.47 (0–115.65)	0.247	NA
Ventilation index at 48 h^a^	0 (0–13.99)	0 (0–0)	<0.001	NA
Renal score at 24 h^a^	0.1 (0–0.1)	0.1 (0–0.2)	0.22	NA
Renal score at 48 h^a^	0.1 (0–0.15)	0.1 (0–0.1)	0.684	NA
VIS at 24 h^a^	14 (11–20)	7 (4–10)	<0.001	NA
VIS at 48 h^a^	10 (6–12)	3 (1–5)	<0.001	NA
VVR at 24 h^a^	29.4 (17.6–35.3)	13 (6–20.12)	<0.001	NA
VVR at 48 h^a^	11 (6.5–26.94)	3.5 (1–6)	<0.001	NA

TOF, tetralogy of Fallot; RACHS-1, risk adjustment for congenital heart surgery; STAT, Society of Thoracic Surgeons—European Association for Cardio-thoracic Surgery; SpO_2_, oxygen saturation; Pre-op, preoperative; CRP, C-reactive protein; PCO_2_, partial pressure of carbon dioxide; RR, respiratory rate; PIP, peak inspiratory pressure; PEEP, positive end-expiratory pressure; VIS, vasoactive inotrope score; VVR, vasoactive-ventilation-renal; OR, odds ratio; NA, not applicable.

^a^ Reported as median (IQR); others are reported as n (%).

**Table 4:** Area under the ROC curve of different demographic and clinical variables for the outcomes of interest (prolonged PICU stay, prolonged length of hospital stay, and prolonged ventilation duration).

Variable	Prolonged PICU stay	Prolonged length of hospital stay	Prolonged ventilation duration
	AUC (95% CI)	AUC (95% CI)	AUC (95% CI)
Age (years)	0.283 (0.202–0.365)*	0.307 (0.215–0.399)*	0.262 (0.165–0.358)*
RACHS-1 score	0.594 (0.506–0.681)**	0.665 (0.571–0.759)**	0.665 (0.571–0.759)**
STAT score	0.563 (0.469–0.658)	0.571 (0.461–0.68)	0.571 (0.461–0.68)
Cross-clamp time	0.569 (0.483–0.656)	0.606 (0.517–0.694)**	0.602 (0.502–0.703)**
Bypass time	0.603 (0.514–0.693)**	0.597 (0.506–0.687)**	0.598 (0.493–0.702)
Pre-op SpO_2_	0.388 (0.293–0.484)**	0.447 (0.348–0.547)	0.348 (0.242–0.453)**
Pre-op creatinine	0.449 (0.359–0.54)	0.508 (0.407–0.61)	0.471 (0.361–0.581)
Creatinine at 24 h	0.462 (0.366–0.558)	0.477 (0.375–0.579)	0.438 (0.326–0.55)
Creatinine at 48 h	0.563 (0.472–0.654)	0.556 (0.463–0.649)	0.517 (0.408–0.626)
Pre-op CRP	0.543 (0.453–0.633)	0.492 (0.396–0.588)	0.579 (0.477–0.681)
Lactate at 1 h	0.571 (0.478–0.665)	0.534 (0.437–0.631)	0.462 (0.352–0.573)
Lactate at 24 h	0.591 (0.498–0.685)	0.584 (0.49–0.677)	0.599 (0.489–0.709)
Lactate at 48 h	0.609 (0.515–0.702)**	0.565 (0.472–0.657)	0.597 (0.493–0.701)
PCO_2_ at 24 h	0.481 (0.379–0.584)	0.443 (0.341–0.544)	0.472 (0.348–0.596)
PCO_2_ at 48 h	0.557 (0.46–0.653)	0.527 (0.429–0.625)	0.488 (0.378–0.598)
RR at 24 h	0.658 (0.559–0.756)*	0.666 (0.566–0.765)*	0.746 (0.635–0.856)*
RR at 48 h	0.575 (0.475–0.676)	0.648 (0.548–0.749)**	0.608 (0.486–0.73)
PIP at 24 h	0.622 (0.526–0.717)**	0.643 (0.546–0.74)**	0.665 (0.561–0.769)**
PIP at 48 h	0.593 (0.494–0.691)	0.591 (0.488–0.694)	0.642 (0.525–0.759)**
PEEP at 48 h	0.592 (0.493–0.69)	0.589 (0.487–0.692)	0.642 (0.525–0.76)**
PEEP at 24 h	0.577 (0.486–0.668)	0.593 (0.5–0.686)**	0.618 (0.518–0.718)**
Ventilation index at 24 h	0.556 (0.463–0.65)	0.594 (0.499–0.688)	0.559 (0.455–0.663)
Renal score at 24 h	0.521 (0.422–0.621)	0.439 (0.337–0.541)	0.448 (0.339–0.558)
VIS at 24 h	0.653 (0.548–0.758)*	0.642 (0.541–0.743)**	0.831 (0.752–0.911)*
VVR at 24 h	0.689 (0.603–0.775)*	0.692 (0.607–0.777)*	0.851 (0.765–0.936)*
Ventilation index at 48 h	0.59 (0.492–0.689)	0.589 (0.486–0.692)	0.658 (0.541–0.775)**
Renal score at 48 h	0.638 (0.544–0.731)**	0.534 (0.438–0.63)	0.515 (0.403–0.628)
VIS at 48 h	0.647 (0.546–0.747)**	0.672 (0.574–0.77)*	0.832 (0.74–0.925)*
VVR at 48 h	0.715 (0.621–0.81)*	0.723 (0.631–0.814)*	0.843 (0.756–0.93)*

RACHS-1, risk adjustment for congenital heart surgery; STAT, Society of Thoracic Surgeons—European Association for Cardio-thoracic Surgery; SpO_2_, oxygen saturation; Pre-op, preoperative; CRP, C-reactive protein; PCO_2_, partial pressure of carbon dioxide; RR, respiratory rate; PIP, peak inspiratory pressure; PEEP, positive end-expiratory pressure; VIS, vasoactive inotrope score; VVR, vasoactive-ventilation-renal; AUC, area under the curve; CI, confidence interval.

*p ≤ 0.001; **p < 0.05.

**Table 5:** Spearman's correlation of different scores for the outcomes of interest (prolonged PICU stay, prolonged length of hospital stay, and prolonged ventilation duration).

	Prolonged PICU stay		Prolonged length of hospital stay		Prolonged ventilation duration	
	Spearman's correlation	*p* value	Spearman's correlation	*p* value	Spearman's correlation	*p* value
**At 24 h**						
Ventilation index	0.127	0.075	0.167	0.018	0.086	0.229
Renal score	0.057	0.429	0.001	0.986	0.023	0.748
VIS	0.241	<0.001	0.287	<0.001	0.391	<0.001
VVR	0.303	<0.001	0.353	<0.001	0.415	<0.001
**At 48 h**						
Ventilation index	0.317	<0.001	0.283	<0.001	0.358	<0.001
Renal score	0.118	0.097	0.069	0.335	0.064	0.369
VIS	0.287	0.001	0.324	<0.001	0.437	<0.001
VVR	0.333	<0.001	0.362	<0.001	0.499	<0.001

VIS, vasoactive inotrope score; VVR, vasoactive-ventilation-renal.

### Length of hospital stay

**Figure 2:**
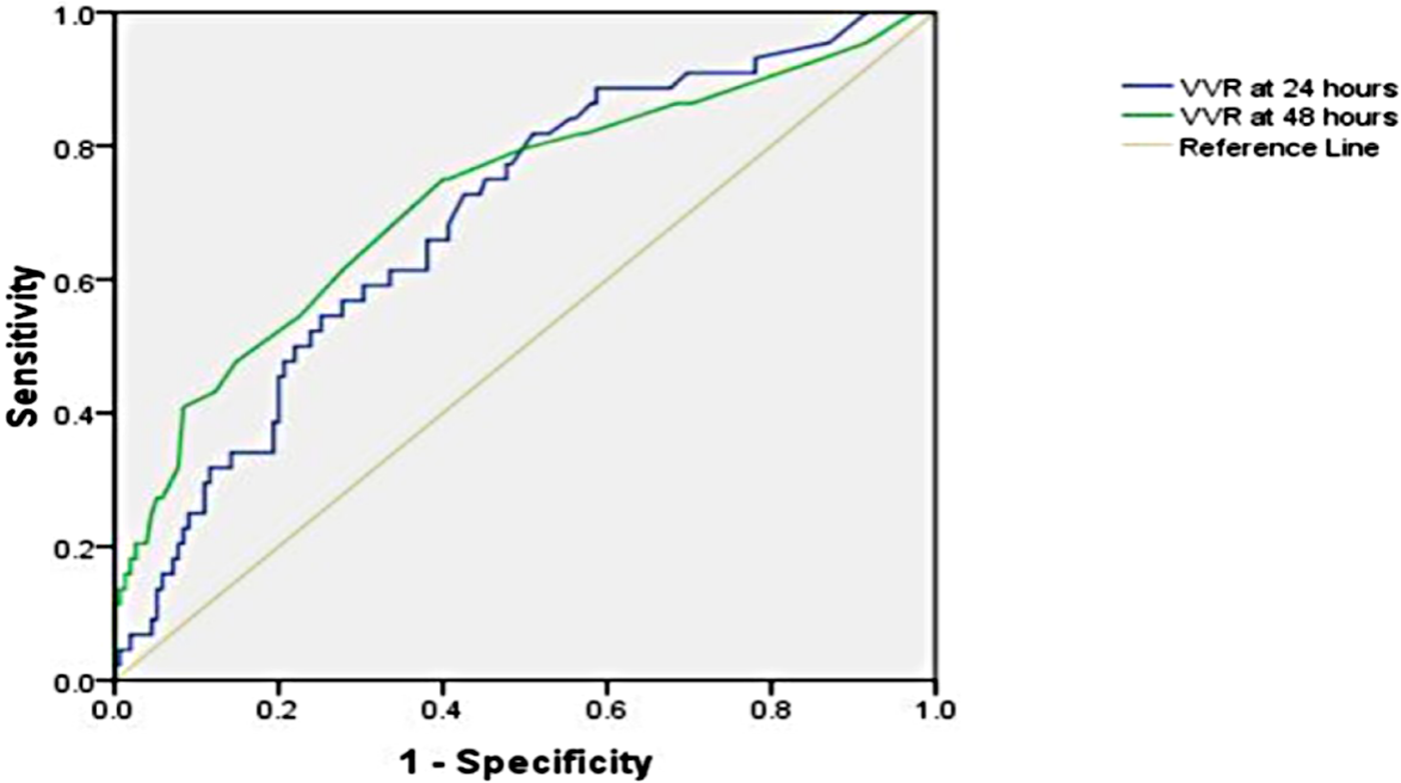
Receiver operating characteristic curve for the prolonged length of hospital stay and vasoactive-ventilation-renal score at 24 h and 48 h postoperatively.

The significant factors that are associated with prolonged LOS (>9 days) are age ≤1 year, cross-clamp time, bypass time, inotrope need at 24 h, inotrope need at 48 h, respiratory rate at 24 h, respiratory rate at 48 h, peak inspiratory pressure at 24 h, peak inspiratory pressure at 48 h, positive end-expiratory pressure at 24 h, positive end-expiratory pressure at 48 h, ventilation index at 24 h, VIS at 24 h, VIS at 48 h, VVR at 24 h, and VVR at 48 h. Other variables are listed in Table 2. When the multivariate regression analysis of the factors with *p* < 0.05 was done, the 48th-h VVR score, PIP at 48 h, and PEEP at 48 h were found to be independent risk factors. For prolonged LOS, at the 24th h, AUC (95% CI) values were higher for the VVR score (*p* < 0.001) (Figure 2) than those for VI, renal score, and VIS. Similarly, at the 48th h, AUC (95% CI) values were higher for the VVR score (*p* < 0.001) (Figure 2) than those for VI, renal score, and VIS. The AUC values for none of the variables were superior to those for the 24th- or 48th-h VVR score in predicting the outcome (Table 4). No significant difference was found in the AUC values for the 24th- and 48th-h VVR score (*z* = 0.91; *p* = 0.361) and for the 48th-h VVR score and VIS (*z* = 1.58; *p* = 0.114) in predicting prolonged LOS. In the correlation analysis, the VVR score predicted the outcome better than the VIS at each measurement point (Table 5). The best cutoff value of 5 for the VVR score at 48 h had a sensitivity of 75% and a specificity of 60% for prolonged LOS.

**Table 2:** Univariate analysis showing factors associated with prolonged length of hospital stay (>9 days).

Variables	>75th percentile (*n* = 44)	≤75th percentile (*n* = 155)	*p* value	OR (95% CI)
Age (≤1 year)	25 (56.8%)	46 (29.7%)	0.001	3.12 (1.57–6.21)
Gender (male)	106 (68.4%)	28 (63.6%)	0.553	0.81 (0.4–1.63)
Diagnosis (TOF)	19 (43.2%)	55 (35.5%)	0.351	1.38 (0.7–2.73)
RACHS-1 (score ≥3)	8 (18.2%)	22 (14.2%)	0.514	1.34 (0.55–3.27)
STAT (score ≥2)	18 (40.9%)	49 (31.6%)	0.249	1.5 (0.75–2.99)
Cross-clamp time (min)^a^	47.5 (36.25–58)	42 (31–53)	0.032	NA
Bypass time (min)^a^	65.5 (57.8–74.5)	62 (49–72)	0.047	NA
Inotrope at 24 h (yes)	44 (100%)	139 (89.7%)	0.025	Cannot be calculated^b^
Inotrope at 48 h (yes)	44 (100%)	134 (86.5%)	0.01	Cannot be calculated^b^
Pre-op SpO_2_ (%)^a^	99 (97–100)	99 (98–100)	0.263	NA
Pre-op creatinine (mg/dL)^a^	0.3 (0.2–0.4)	0.3 (0.2–0.4)	0.864	NA
Creatinine at 24 h (mg/dL)^a^	0.4 (0.3–0.5)	0.4 (0.3–0.5)	0.633	NA
Creatinine at 48 h (mg/dL)^a^	0.4 (0.3–0.5)	0.4 (0.3–0.5)	0.242	NA
Pre-op CRP (mg/L)^a^	5 (5–5)	5 (5–5)	0.835	NA
Lactate at 1 h (mmol/L)^a^	2.51 (1.83–3.49)	2.31 (1.79–3.3)	0.463	NA
Lactate at 24 h (mmol/L)^a^	1.55 (1.18–1.85)	1.35 (1.03–1.79)	0.081	NA
Lactate at 48 h (mmol/L)^a^	1.14 (0.96–1.52)	1.08 (0.89–1.32)	0.191	NA
PCO_2_ at 24 h (mmHg)^a^	36.3 (33.1–40.1)	37.4 (34.9–40.4)	0.267	NA
PCO_2_ at 48 h (mmHg)^a^	38.5 (36.0–41.8)	37.9 (35.1–40.7)	0.584	NA
RR at 24 h^a^	26.5 (0–35)	16 (0–25)	<0.001	NA
RR at 48 h^a^	21.5 (0–27.3)	0 (0–20)	0.001	NA
PIP at 24 h^a^	18 (0–20)	14 (0–18)	0.002	NA
PIP at 48 h^a^	0 (0–0)	0 (0–0)	<0.001	NA
PEEP at 48 h^a^	5 (0–5)	5 (0–5)	0.027	NA
PEEP at 24 h^a^	0 (0–0)	0 (0–0)	<0.001	NA
Ventilation index at 24 h^a^	67.1 (0–148.1)	13.13 (0–110.5)	0.048	NA
Ventilation index at 48 h^a^	0 (0–0)	0 (0–0)	0.088	NA
Renal score at 24 h^a^	0.1 (0–0.18)	0.1 (0–0.2)	0.215	NA
Renal score at 48 h^a^	0.1 (0–0.1)	0.1 (0–0.1)	0.423	NA
VIS at 24 h^a^	11 (5.5–14)	7 (4–10)	0.004	NA
VIS at 48 h^a^	6 (1–10)	3 (1–5)	<0.001	NA
VVR at 24 h^a^	21.5 (13.5–29.8)	13 (6–20.8)	<0.001	NA
VVR at 48 h^a^	7 (4.3–13.8)	3 (1–6)	<0.001	NA

TOF, tetralogy of Fallot; RACHS-1, risk adjustment for congenital heart surgery; STAT, Society of Thoracic Surgeons—European Association for Cardio-thoracic Surgery; SpO_2_, oxygen saturation; Pre-op, preoperative; CRP, C-reactive protein; PCO_2_, partial pressure of carbon dioxide; RR, respiratory rate; PIP, peak inspiratory pressure; PEEP, positive end-expiratory pressure; VIS, vasoactive inotrope score; VVR, vasoactive-ventilation-renal; OR, odds ratio; NA, not applicable.

^a^ Reported as median (IQR); others are reported as n (%).

^b^ Cannot be calculated—The number of children in the >75th percentile group without inotrope at 24 and 48 h is 0. Therefore, the denominator for the calculation of OR is 0, giving an infinity value for OR.

### Ventilation duration

**Figure 3:**
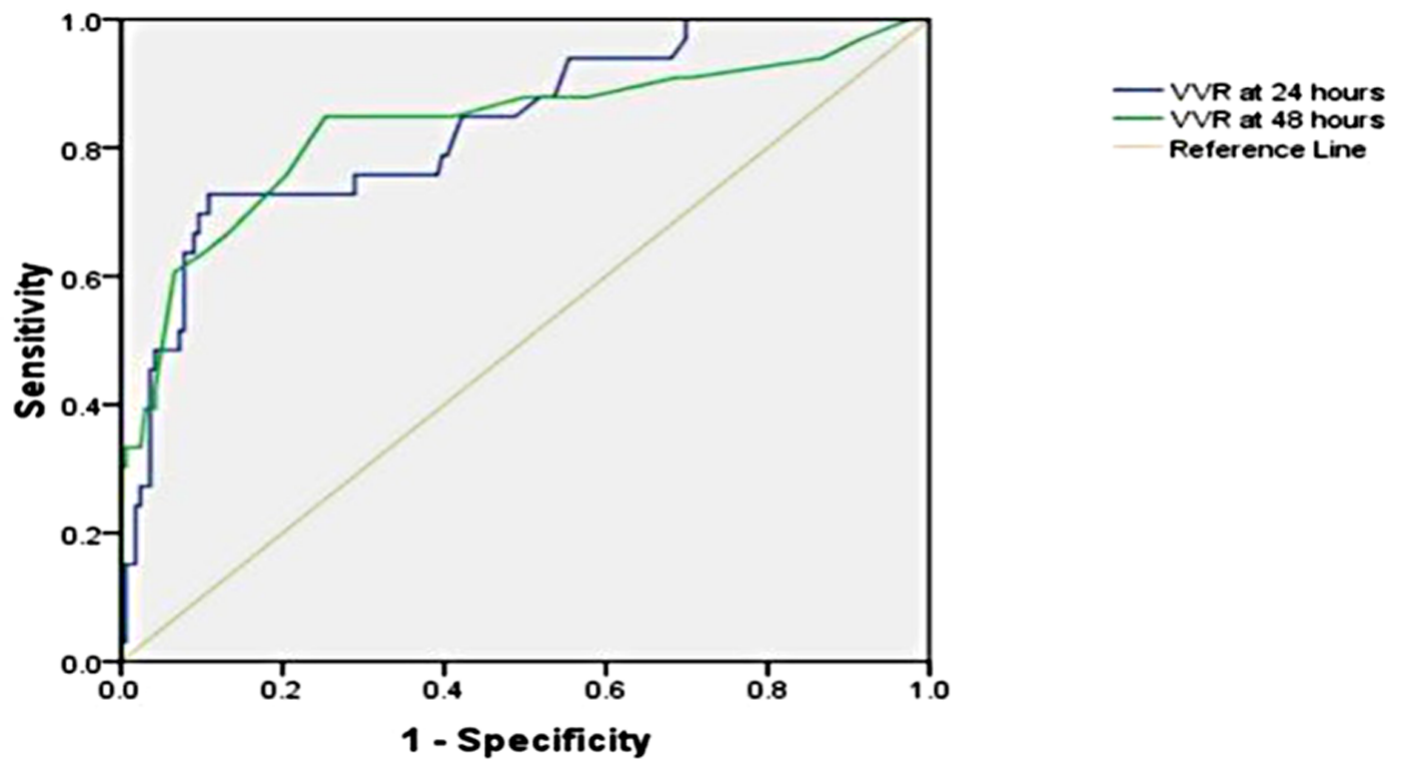
Receiver operating characteristic curve for the prolonged ventilation duration and vasoactive-ventilation-renal score at 24 h and 48 h postoperatively.

The significant factors associated with ventilation duration (>20 h) are age ≤1 year, RACHS-1 score ≥3, inotrope need at 48 h, preoperative SpO_2_, respiratory rate at 24 h, peak inspiratory pressure at 24 h, peak inspiratory pressure at 48 h, positive end-expiratory pressure at 24 h, positive end-expiratory pressure at 48 h, ventilation index at 48 h, VIS at 24 h, VIS at 48 h, VVR at 24 h, and VVR at 48 h. Other variables are listed in Table 3. When the multivariate regression analysis of the factors with *p* < 0.05 was done, age ≤1 year, RACHS-1 score ≥3, and 48th-h VVR score were found to be independent risk factors. For prolonged ventilation duration, at the 24th h, AUC (95% CI) values were higher for the VVR score (*p* < 0.001) (Figure 3) than those for VI, renal score, and VIS. Similarly, at the 48th h, AUC (95% CI) values were higher for the VVR score (Figure 3) than those for VI, renal score, and VIS. The AUC values for none of the variables were superior to those for the 24th- or 48th-h VVR score in predicting the outcome (Table 4). No significant difference was found in the AUC values for 24th- and 48th-h VVR scores (*z* = 0.044; *p* = 0.965) and for the 48th-h VVR score and VIS (*z* = 0.83; *p* = 0.409) in predicting prolonged ventilation duration. In the correlation analysis, the VVR score predicted the outcome better than the VIS at each measurement point (Table 5). The best cutoff value of 5.5 for the VVR score at 48 h had a sensitivity of 85% and a specificity of 75% for prolonged mechanical ventilation.

## Discussion

Our study further validated previous studies on VVR scores while providing new comparison points with reference to simpler scores (11, 14–17). We have been able to establish a correlation between the VVR score and prolonged PICU stay, better than other scoring systems. We also demonstrated the predictive value of the VVR score on the length of hospital stay and ventilation duration. Most of our pediatric patients were boys (67.3%) under 6 years of age, with 35.7% under 1 year, and having ventricular septal defect (46.2%) and tetralogy of Fallot (37.2%). This heterogenous group increased the strength of the study.

Compared to all other parameters, the VVR score at 48 h postoperation was found to best correlate with prolonged PICU stay (>96.5 h), prolonged length of hospitalization (>9 days), and prolonged ventilation duration (>20 h), with the greatest AUC-ROC (0.715, 0.723, 0.843, respectively). This indicates the significant contribution of ventilation and renal function to patients’ postcardiac surgery outcomes. Alam et al., in their study on 1,097 patients, found a similar correlation between LOS and mortality (13). While the VIS also correlated better at 48 h than at 24 h postoperation, it was found to be poorer than the VVR score at both times. A study by Scherer et al. deduced that the VVR score better represented LOS than VIS (17). Miletic et al. proposed using this score and, in a prospective study, showed that 48th-h VVR score predicted both LOS and prolonged mechanical ventilation duration better than VIS and serum lactate (11, 16). A study conducted by Havan et al. found the 48-h VVR score to be an effective predictor of the LOS and duration of mechanical ventilation in children postcardiac surgery, as found in our study (28). Another retrospective study conducted by Ozturk et al. found that the VVR score at 48 h is a useful tool in determining the duration of ICU stay and hospital mortality in children postcardiac surgery (18).

There was no significant association between any of the three outcomes and the renal score at either 24-h or 48-h postoperation. VI at 24 h postoperation was found to be associated with a prolonged hospital stay but not with the other outcomes; however, at 48 h postoperation, the score was found to be mildly associated with all three outcomes.

Out of the other factors analyzed, RACHS-1 score ≥ 3, STAT score ≥ 2, and cross-clamp time were not significant factors in determining the length of PICU stay. The RACHS-1 score was calculated to determine the risk of hospital mortality postcardiac surgery for congenital heart disease in pediatric patients. RACHS-1 categorizes several surgical palliative or corrective procedures for congenital heart disease (CHD) into six categories according to operative risk mortality (29). It has since been used as a predictor of perioperative recovery in pediatric patients. A study showed that RACHS-1 score >4 is an effective factor for prolonged ICU stay (18). On the other hand, lactate at 24 and 48 h and bypass time showed a statistically significant association. Similar results were seen for longer hospitalization, with the addition of cross-clamp time also being significant. Other studies showed a relation between RACHS category ≥3 and prolonged LOS (13, 30, 31). RACHS-1 score ≥ 3, along with the previous factors, was found to be significant for prolonged ventilation duration. Even though these factors are relatively easier to calculate and obtain, the correlation of the VVR score was found to be the strongest, thus enabling it to be a better indicator of outcomes in children postcardiac surgery. Boethig et al. conducted a study to analyze the relationship of the RACHS-1 score with mortality and LOS. They found that while RACHS-1 is valuable in predicting LOS, its use for individual prediction is limited due to marked intraclass scattering of the length of stay times observed in their study (32). The STAT score was designed to analyze the risk for mortality associated with congenital heart surgery procedures. STAT score ≥3 was found to be associated with 30-day- and 1-year-mortality and 1-year readmission postcardiac surgery in a study conducted by Nunes et al. (33). The STAT score has not been found to be significantly associated with PICU LOS postcardiac surgery, and studies on the same are limited. Gaies et al. found that a higher STAT category was associated with a longer duration of postoperative mechanical ventilation, although our study did not produce similar results (34).

The VVR score is a bedside method with several other advantages including ease of calculation and cost-effectiveness, and it is a strong predictor of postcardiac surgery outcomes. These advantages may lead to a preference for the VVR score over newer experimental biomarkers in this population. However, VVR is not without its limitations. VVR calculates the renal dysfunction parameter using the difference in preoperative and postoperative creatinine values, which may be an inaccurate representation (13, 35, 36). This is because serum creatinine widely varies depending on age and often underrepresents the true effect of kidney injury (13). Alternative parameters to calculate renal dysfunction such as the difference in the percentage of estimated glomerular filtration should be evaluated for possible use in the future. The use of VI is not reliable in children requiring mechanical ventilation, and further evaluation to improve its accuracy and the usefulness of VI using plateau pressure (VI-PLAT) should be considered (13, 35). VVR has not been verified with complex measurement methods including logistic organ dysfunction score, pediatric risk of mortality III score, and pediatric index of mortality II score (18, 37, 38). However, VVR is much easier to calculate than these scores.

The major limitation of this study is that it is a single-center study. However, the center is in a developing country, thus helping validate the score in this region. Other limitations include the smaller sample size and the limited number of cardiac defects analyzed. Only a selective pediatric population [mean age 2 (0.8–5) years] undergoing elective cardiovascular reparative surgery was included in the study. This study gives a good comparison of various other outcome measures.

## Data Availability

The original contributions presented in the study are included in the article/**Supplementary Material**, further inquiries can be directed to the corresponding author.
