# Supplementary material for: Vasoactive-ventilation-renal score and outcomes in infants and children after cardiac surgery
Source: Front Pediatr. 2023 Feb 20;11:1086626. doi: 10.3389/fped.2023.1086626 (PMC9986414; doi:10.3389/fped.2023.1086626)
Supplement: Supplementary file 1 [file Table1.docx]

**Supplementary table 1: Demographic profile of the patients undergoing cardiovascular surgery (n=199)**

| Variables | | N (%) or Median (IQR) |
| --- | --- | --- |
| Age group [years] | ≤1 | 71 (35.7%) |
|  | >1 - 6 | 94 (47.2%) |
|  | >6 - 10 | 19 (9.5%) |
|  | >10 - 18 | 15 (7.5%) |
| Age [years] | | 2 (0.75 - 5) |
| Weight [kg] | | 9.3 (6 - 16) |
| Gender [Male] | | 134 (67.3%) |
| Diagnosis | Atrial septal defect | 33 (16.6%) |
|  | Ventricular septal defect | 92 (46.2%) |
|  | Tetralogy of fallot | 74 (37.2%) |
| RACHS 1 score | 1 | 35 (17.6%) |
|  | 2 | 134 (67.3%) |
|  | 3 | 30 (15.1%) |
| STAT score | 1 | 132 (66.3%) |
|  | 2 | 67 (33.7%) |
| Cross clamp time [minutes] | | 44 (33 - 54) |
| Bypass time [minutes] | | 63 (51 - 72) |
| Inotrope at 24 hrs [Yes] | | 183 (92%) |
| Dopamine at 24 hrs [Yes] | | 92 (46.2%) |
| Epinephrine at 24 hrs [Yes] | | 123 (61.8%) |
| Milrinone at 24 hrs [Yes] | | 176 (88.4%) |
| Norepinephrine at 24 hrs [Yes] | | 37 (18.6%) |
| Inotrope at 48 hrs [Yes] | | 178 (89.4%) |
| Dopamine at 48 hrs [Yes] | | 46 (23.1%) |
| Epinephrine at 48 hrs [Yes] | | 63 (31.7%) |
| Milrinone at 48 hrs [Yes] | | 174 (87.4%) |
| Norepinephrine at 48 hrs [Yes] | | 26 (13.1%) |
| Pre-op SpO2 | | 99 (98 - 100) |
| Pre-op creatinine | | 0.3 (0.2 - 0.4) |
| Creatinine at 24 hrs | | 0.4 (0.3 - 0.5) |
| Creatinine at 48 hrs | | 0.4 (0.3 - 0.5) |
| Pre-op CRP | | 5 (5 - 5) |
| Lactate at 1 hr | | 2.34 (1.8 - 3.39) |
| Lactate at 24 hr | | 1.4 (1.07 - 1.8) |
| Lactate at 48 hr | | 1.1 (0.9 - 1.39) |
| PCO2 at 24 hrs | | 37.3 (34.2 - 40.2) |
| PCO2 at 48 hrs | | 38 (35.1 - 40.85) |
| Respiratory rate at 24 hrs | | 18 (0 - 28) |
| Respiratory rate at 48 hrs | | 15 (0 - 22) |
| PIP at 24 hrs | | 15 (0 - 18) |
| PIP at 48 hrs | | 0 (0 - 0) |
| PEEP at 48 hrs | | 0 (0 - 0) |
| PEEP at 24 hrs | | 5 (0 - 5) |
| Ventilation index at 24 hrs | | 22.7 (0 - 118.66) |
| Ventilation index at 48 hrs | | 0 (0 - 0) |
| Renal Score at 24 hrs | | 0.1 (0 - 0.2) |
| Renal Score at 48 hrs | | 0.1 (0 - 0.1) |
| VIS at 24 hrs | | 7 (4 - 11) |
| VIS at 48 hrs | | 4 (1 - 6) |
| VVR at 24 hrs | | 14.72 (7.2 - 23.39) |
| VVR at 48 hrs | | 4 (1 - 7) |
| Length of stay in hospital [days] | | 8 (7 - 9) |
| Picu stay [hours] | | 80 (69.5 - 96.5) |
| Ventilation [hours] | | 16 (14 - 20) |
| Inotrope duration [hours] | | 48 (48 - 72) |

RACHS-1: Risk Adjustment for Congenital Heart Surgery; STAT: The Society of Thoracic Surgeons-European Association for Cardio-Thoracic Surgery; min: minute; SpO2: Oxygen saturation; Pre-op: Pre-operative; mg: milligram; l: litre; dl: decilitre; CRP: C-reactive protein; PCO2: partial pressure of carbon dioxide; mmol: millimole; mmHg: millimetre of mercury; hrs: hours; RR: Respiratory rate; PIP: peak inspiratory pressure; PEEP: Positive end-expiratory pressure; VIS: vasoactive inotrope score; VVR: Vasoactive-Ventilation-Renal; OR: Odds ratio; NA: Not applicable
